# Multi-Task Deep Learning for Sex and Age Estimation from Panoramic Radiographs in a Brazilian Young Population

**DOI:** 10.1016/j.identj.2025.109381

**Published:** 2026-01-27

**Authors:** Matheus L. Oliveira, Su Yang, Matheus Sampaio-Oliveira, Deborah Queiroz Freitas, Francisco Haiter-Neto, Sang Heon Lim, Jiyong Han, Sujeong Kim, Jun-Min Kim, Won-Jin Yi, Min-Suk Heo

**Affiliations:** a Department of Oral Diagnosis, Piracicaba Dental School, University of Campinas, Piracicaba, São Paulo, Brazil; b Department of Oral and Maxillofacial Surgery, Houston Methodist Research Institute, Houston, Texas, USA; c Interdisciplinary Program in Bioengineering, Seoul National University, Gwanak-gu, Seoul, Republic of Korea; d Department of Electronics and Information Engineering, Hansung University, Seongbuk-gu, Seoul, Republic of Korea; e Department of Oral and Maxillofacial Radiology and Dental Research Institute, Seoul National University, Jongno-gu, Seoul, Republic of Korea

**Keywords:** Forensic dentistry, Age estimation, Sex estimation, Panoramic radiography, Multi-task deep learning

## Abstract

**Introduction and aims:**

Accurate estimation of age and sex is crucial in forensic and clinical contexts, however conventional methods are subjective and time-consuming. Panoramic radiographs offer valuable data for automated analysis. Therefore, the aim of this study was to present and evaluate a multi-task deep learning framework based on ForensicNet for simultaneous estimation of chronological age and classification of sex using panoramic radiographs of the Brazilian young population aged 5-15 years.

**Methods:**

A total of 2200 high-resolution panoramic radiographs were retrospectively collected, balanced by age and sex. After applying strict inclusion/exclusion criteria, the images were randomly split into training (1320), validation (440), and test (440) sets. A multi-task DL model based on EfficientNet-B3 was implemented with task-specific branches incorporating Convolutional Block Attention Modules (CBAM) to predict age and sex. The model was trained end-to-end using a weighted multi-task loss (α = 0.3 for age, β = 0.7 for sex) and evaluated against five benchmark architectures. Grad-CAM was used for model interpretability.

**Results:**

The proposed ForensicNet outperformed all baseline models, achieving the lowest mean absolute error and highest coefficient of determination in age prediction, and highest accuracy and area under the curve in sex classification. Grad-CAM visualisations confirmed the model’s focus on anatomically relevant areas. Ablation studies showed that removing CBAM or altering task weights reduced performance.

**Conclusions:**

The proposed ForensicNet-based multi-task deep learning model demonstrated robust performance in both chronological age estimation and sex classification using panoramic radiographs from young Brazilian individuals, supporting its potential forensic and clinical applicability.

**Clinical relevance:**

This framework may assist forensic experts and clinicians by providing fast, objective and reproducible estimations of age and sex from routinely acquired panoramic radiographs, potentially improving identification processes in forensic and pediatric contexts.

## Introduction

Chronological age estimation and sex classification play a crucial role in forensic science and individual identification, aiding in the narrowing of potential suspects during investigations.^1^, ^2^, ^3^ Traditionally, methods such as forensic deoxyribonucleic acid (DNA) testing and morphological assessments of durable tissues have been widely utilised.^3^^,^^4^ Among these, the assessment of dental structures has gained increasing attention as an accurate, efficient and reliable approach.^5^, ^6^, ^7^

Multiple dental age estimation methods for children and adolescents rely on panoramic radiographs, as this imaging modality offers important insights into dental and skeletal maturity.^2^^,^^8^ However, these traditional approaches are typically labour-intensive, time-consuming and susceptible to human error and interobserver variance.^5^^,^^9^ As a result, there is a growing need for an automated and reliable method capable of efficiently estimating chronological age and classifying sex from panoramic radiographs.

Several studies have explored deep learning (DL)-based methods, an area within artificial intelligence (AI), for the relevant task of estimating chronological age and classifying sex using panoramic radiographs.^10^^,^^11^ These studies report promising results, with high accuracy in sex classification, particularly in adults and low errors in age estimation across diverse age ranges.^10^^,^^11^ Collectively, these findings highlight the potential of AI-driven solutions to improve the efficiency and reliability of forensic assessments based on dental imaging.^6^

Most existing studies in this field focus on the independent prediction of age or sex, rather than addressing both simultaneously.^10^, ^11^, ^12^, ^13^, ^14^, ^15^, ^16^ Furthermore, many of these investigations are limited by imbalanced datasets, often characterised by the over-representation of specific age groups or one sex.^6^ A recent study introduced ForensicNet which was specifically developed to be a multi-task DL framework for simultaneous estimation of chronological age and classification of sex from panoramic radiographs.^17^ This framework present some relevant characteristics, such as improving efficiency and eliminating the need for separate models, the incorporation of a convolutional block attention module (CBAM) enhancing the ability to capture long-range anatomical relationships, resulting in more robust predictions and the use of a weighted multi-task, ensuring balanced optimisation.^17^

However, because the model was originally tested only in a South Korean population, it is essential to evaluate its performance in populations with different ethnic backgrounds and across varying age ranges to ensure its broader applicability and generalizability.

Therefore, the aim of this study was to present and evaluate the performance of a multi-task DL framework based on ForensicNet^17^ for the simultaneous estimation of chronological age and classification of sex from panoramic radiographs of children and young adolescents aged 5-15 years in the Brazilian population.

## Materials and methods

This retrospective study was approved by the local Research Ethics Committee (protocol CAAE 85107024.9.0000.5418) and conducted in accordance with the principles of the Declaration of Helsinki.

### Data collection and curation

A database of panoramic radiographs from Brazilian patients aged 5-15 years, acquired between 2019 and 2024, was used (Supplemental Figure S1). All panoramic radiographs of the database were acquired using the OP300 Maxio system (Instrumentarium Dental, Tuusula, Finland) at a tube voltage of 66 kVp and tube current of 10 mA.

Inclusion criteria comprised panoramic radiographs from Brazilian individuals demonstrating adequate anatomical coverage, proper patient positioning and acceptable image quality, free from artefacts such as earrings or removable prostheses and without issues related to abnormal density, contrast or noise. Exclusion criteria comprised radiographs associated with edentulous patients, individuals who had undergone orthognathic surgery, patients with maxillofacial reconstructions or those presenting extensive intraosseous lesions. Each radiograph meeting these criteria was annotated with the patient’s chronological age and sex retrieved from the electronic medical record system.

The panoramic radiographs were consecutively selected until a balanced distribution of 200 radiographs per age group was achieved, comprising 100 from males and 100 from females within each group, totaling 2200 panoramic radiographs. The selected panoramic radiographs were anonymised and randomly divided into training, validation and test sets using a 3:1:1 ratio, resulting in 1320 panoramic radiographs for training, 440 for validation and 440 for testing. To ensure consistency, each subset preserved the same distribution of age range (5-15 years) and sex (female and male). All panoramic radiographs were in high-resolution 8-bit Joint Photographic Experts Group (JPG) format, with heights ranging from 976 to 1468 pixels and widths ranging from 1976 to 2988 pixels. To train the multi-task DL framework, all panoramic radiographs were resized to 480 × 960 pixels.

### Multi-task deep learning architecture

**Fig. 1:**
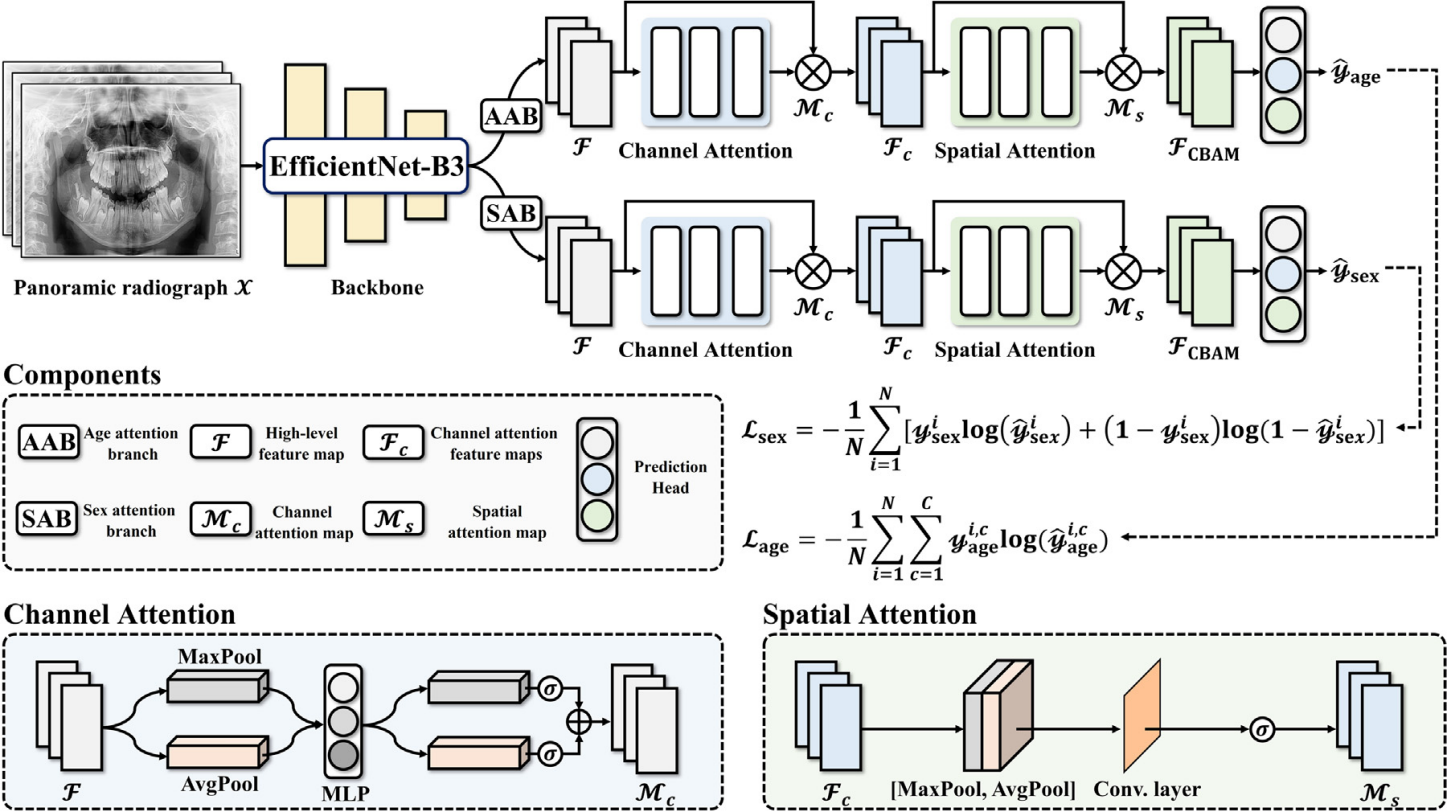
Proposed multitask deep learning framework, ForensicNet, based on EfficientNet-B3 with chronological age and sex attention branches. Each branch includes a convolutional block attention module with channel and spatial mechanisms.

A multi-task DL framework based on ForensicNet^17^ was used to simultaneously estimate chronological age and classify sex from the selected panoramic radiographs (Figure 1). This framework integrates an EfficientNet-B3 backbone^18^ for feature extraction with two task-specific branches: one for age estimation and the other for sex classification. Each branch uses a CBAM^19^ followed by dedicated prediction heads.

The model was trained end-to-end using a weighted multi-task loss function that combines binary cross-entropy for sex classification and categorical cross-entropy for age estimation. To accommodate categorical age prediction, the output activation function of the age attention branch was modified from the original ForensicNet design. The input panoramic radiograph $X\in R^{h\;\times w\times \;1}$, where *h* and *w* represent height and width, was processed by the EfficientNet-B3 backbone to extract high-level feature maps F, as follows:

$$F=Backbone\left(X\right)\in R^{h\;\times w\times d},$$

where *h, w* and *d* denote the height, width and depth dimensions of the output feature maps, with values of 15, 30 and 1536, respectively. The F was fed into two separate task-specific attention branches: the sex attention branch (SAB) and the age attention branch (AAB). Each incorporates CBAM to capture variations in size and shape according to the sex and chronological age in children and young adolescents.^17^ The channel attention map $M_{c}\in R^{1\;\times \;1\;\times d}$ was extracted from F as follows:

$$M_{c}=\sigma \left(W_{0}\left(\text{AvgPool}\left(F\right)\right)\right)+\sigma \left(W_{0}\left(\text{MaxPool}\left(F\right)\right)\right),$$

where *σ, W*_0_, AvgPool and MaxPool denote the sigmoid activation function, a shared multilayer perceptron, a global average-pooling layer and a global max-pooling layer, respectively. The depth dimension was set as 1536. The channel attention feature maps $F_{c}$ were acquired by:

$$F_{c}=M_{c}\;\left(F\right)⊙F$$

where ⊙ denotes element-wise multiplication. After the channel attention, the spatial attention map $M_{c}\in R^{h\;\times w\times \;1}$ was computed along the spatial dimension of $F_{c}$. The spatial dimensions *h* and *w* are 15 and 30, respectively. First, average-pooling and max-pooling were applied across the channels of $F_{c}$; these pooled features were then concatenated and passed through a 7 × 7 convolutional layer $f^{7\;\times \;7}$. The spatial attention map $M_{s}$ was acquired by:

$$M_{s}=\sigma \left(f^{7\;\times \;7}\left(\left[\text{AvgPool}\left(F_{c}\right),\text{MaxPool}\left(F_{c}\right)\right]\right)\right)$$

where *σ*, AvgPool and MaxPool denote the sigmoid activation function, global average-pooling layer and global max-pooling layer, respectively. The final output of the CBAM, $F_{\text{CBAM}}$, was defined as:

$$F_{\text{CBAM}}=M_{s}\;\left(F_{c}\right)⊙F_{c},$$

Although age is inherently a continuous variable, ForensicNet formulates age estimation as a multi-class classification problem over a fixed age range (eg, 5-15 years). In the age prediction head of the AAB, the age feature vector $z_{\text{age}}\in R^{d}$ is extracted from $F_{\text{CBAM}}$ using global average pooling and is then passed through a fully connected layer $W_{\text{age}}$ with a Softmax activation function, as follows:

$${\hat{y}}_{\text{age}}=\mu \left(W_{\text{age}}\cdot z_{\text{age}}+b_{\text{age}}\right)\in R^{C},$$

where $b_{\text{age}}$ is the bias term of the age prediction head, *C* is the number of discrete age classes, *μ* denotes the SoftMax activation function and ${\hat{y}}_{\text{age}}$ represents the predicted age probability distribution. Similarly, in the sex prediction head of the SAB, the sex feature vector $z_{\text{sex}}\in R^{d}$ is acquired from $F_{\text{CBAM}}$ via global average pooling and processed through a fully connected layer $W_{\text{sex}}$ with a Sigmoid activation function, as follows:

$${\hat{y}}_{\text{sex}}=\sigma \left(W_{\text{sex}}\cdot z_{\text{sex}}+b_{\text{sex}}\right),\;{\hat{y}}_{\text{sex}}\in \left[0,1\right],$$

where $b_{\text{sex}}$ is the corresponding bias term, σ denotes the Sigmoid activation function and ${\hat{y}}_{\text{sex}}$ is the predicted probability of the sex classification. The prediction heads in the SAB and AAB transform task-specific feature vectors into final outputs for sex and chronological age estimation, respectively.

To enhance the multi-task DL framework interpretability, gradient-weighted class activation mapping (Grad-CAM) was applied.^20^ Grad-CAM generates heatmaps that highlight the regions of panoramic radiographs most influential in the model’s sex and age predictions. This visualisation enables assessment of whether the network focuses on anatomically relevant structures, such as the teeth and surrounding bone structures, thereby ensuring alignment between predictions and meaningful anatomical features.

### Loss function with task-specific weighting

For network training, a weighted multi-task loss function $L_{\text{WML}}$ was designed to jointly optimise sex and age classification in ForensicNet. This loss function combines the categorical cross-entropy loss $L_{\text{age}}$ for age classification and the binary cross-entropy loss $L_{\text{sex}}$ for sex classification. Given a one-hot encoded age ground truth $y_{\text{age}}^{i}\in {\left\{0,1\right\}}^{C}$ and predicted age probabilities ${\hat{y}}_{\text{age}}^{i}\;\in {\left[0,1\right]}^{C}$, the age classification is defined as:

$$L_{\text{age}}=\frac{1}{N}\sum_{i=1}^{N}\sum_{c=1}^{C}y_{\text{age}}^{i,c}\log\left({\hat{y}}_{\text{age}}^{i,c}\right),$$

where $y_{\text{age}}^{i}$ and ${\hat{y}}_{\text{age}}^{i,c}$ denote the ground truth and predicted probability, respectively, for the i-th image belonging to class *c* and *N* is the total number of panoramic radiographs. For binary sex classification, with a ground truth label $y_{\text{sex}}^{i}\in \left\{0,1\right\}$ and predicted probability ${\hat{y}}_{\text{sex}}^{i}\;\in \left[0,1\right]$, the loss is defined as:

$$L_{\text{sex}}=\frac{1}{N}\sum_{i=1}^{N}\left[y_{\text{sex}}^{i}\log\left({\hat{y}}_{\text{sex}}^{i}\right)+\left(1-y_{\text{sex}}^{i}\right)\log\left(1-{\hat{y}}_{\text{sex}}^{i}\right)\right]$$

The total weighted multi-task loss is then defined as:

$$L_{\text{WML}}=\alpha L_{\text{age}}+\beta L_{\text{sex}},$$

where *α* and *β* are task-balancing weights. Since minimizing $L_{\text{sex}}$ was empirically found to be more challenging than minimizing $L_{\text{age}}$ in the multi-task setting, asymmetric weights were applied. Based on experimental tuning, the values were set to *α* = 0.3 and *β* = 0.7. The training setup is provided in the supplementary materials.

### Performance evaluation

ForensicNet, the model specifically trained for this study, was applied to the tasks of chronological age estimation and sex classification alongside five well-established architectures: VGG16,^21^ MobileNet v2,^22^ ResNet101,^23^ InceptionResNet v2^24^ and DenseNet121.^25^ To ensure a fair comparison, all models were implemented in the same computing environment and subjected to identical data augmentation protocols.

The performance of each model on chronological age estimation was assessed using four key metrics: mean absolute error (MAE), maximum deviation (MD), coefficient of determination (R^2^) and successful estimation rate (SER). For sex classification, the evaluation relied on accuracy (ACC), specificity (SPE), sensitivity (SEN), area under the receiver operating characteristic curve (AUC) and Brier score (BR). Specifically, MAE quantifies the average absolute difference between estimated and actual ages. MD denotes the maximum absolute deviation between estimated and actual ages relative to their mean. R^2^ measures the proportion of variance in the dependent variable explained by the model. SER represents the proportion of samples with absolute age estimation errors within 1-, 2-, 3-, 4- and 5-year thresholds. ACC denotes the proportion of correctly classified instances, SPE and SEN measure the ability to correctly identify true negatives and true positives, respectively, AUC assesses the discriminatory capacity of a model between sex categories and BR represents the mean squared difference between predicted probabilities and actual binary outcomes, thereby measuring the accuracy of probabilistic predictions. MAE values were compared across different DL networks using analysis of variance (ANOVA), followed by Tukey’s post-hoc test for multiple comparisons. ACC values were compared using the McNemar test. A significance level of 5% (α = 0.05) was adopted for all tests. The remaining metrics were assessed descriptively. Additional information on the performance evaluation is provided in the supplementary materials.

## Results

The overall performance of the DL networks for both chronological age estimation and sex classification is summarised in Table 1. ForensicNet demonstrated superior performance across multiple metrics, outperforming all other models in terms of MAE and R^2^ for chronological age estimation, as well as ACC and SEN for sex classification. While MobileNet v2 yielded the lowest MD in age estimation, InceptionResNet v2 achieved the highest SPE in sex classification.

**Table 1:** Performance of the deep learning networks for both chronological age estimation and sex classification.

Deep learning network	Chronological age			Sex		
	MAE (↓)	MD (↓)	R^2^ (↑)	ACC (↑)	SPE (↑)	SEN (↑)
VGG16	0.77 ± 0.83 ab	6.23	0.87	0.78 b	0.74	0.81
MobileNet v2	0.93 ± 0.86 a	**5.07**	0.84	0.83 b	0.81	0.84
ResNet101	0.77 ± 0.89 ab	5.23	0.86	0.88 a	0.91	0.86
InceptionResNet v2	0.76 ± 0.89 b	6.24	0.86	0.79 b	**0.96**	0.63
DenseNet121	0.72 ± 0.83 bc	5.28	0.88	0.89 a	0.91	0.87
ForensicNet	**0.57 ± 0.71** c	6.43	**0.92**	**0.90 a**	0.91	**0.89**

ACC, accuracy; MAE, mean absolute error (±standard deviation); MD, maximum deviation; R², coefficient of determination;SPE, specificity; SEN, sensitivity.

Upward (↑) and downward (↓) arrows indicate the desired direction of improvement.

Bold values indicate the best performance for each metric across networks.

MAE and ACC values followed by different letters differ significantly among deep learning networks (*P* < .05)

When assessing the success estimation rate for chronological age prediction across varying error thresholds (1-, 2-, 3-, 4- and 5-year margins), ForensicNet consistently outperformed the other classification networks (Table 2). Higher success estimation rates indicate greater overall prediction accuracy and improved reliability in estimating ages within narrower error bounds.

**Table 2:** Successful estimation rate (in percentage) of chronological age using different deep learning networks within 1-, 2-, 3-, 4-, and 5-year errors margins.

Deep learning network	Successful estimation rate				
	<1.0	<2.0	<3.0	<4.0	<5.0
VGG16	84.72	97.04	99.55	99.72	99.72
MobileNet v2	78.63	96.59	99.09	99.54	99.72
ResNet101	84.32	95.45	98.64	99.72	99.72
InceptionResNet v2	82.27	95.31	99.55	99.72	99.72
DenseNet121	82.27	95.91	99.55	99.72	99.72
ForensicNet	**93.63**	**98.86**	**99.77**	**99.77**	**99.77**

Bold values indicate the best performance for each metric across networks.

Supplemental Table S1 presents the mean absolute error of chronological age estimation for each age between 5 and 15 years across the different classification networks. ForensicNet achieved the lowest mean absolute error in four age groups (7, 10, 11 and 14), followed by DenseNet121 in three age groups, MobileNet v2 and ResNet101 in two age groups each and InceptionResNet v2 in one age group.

**Fig. 2:**
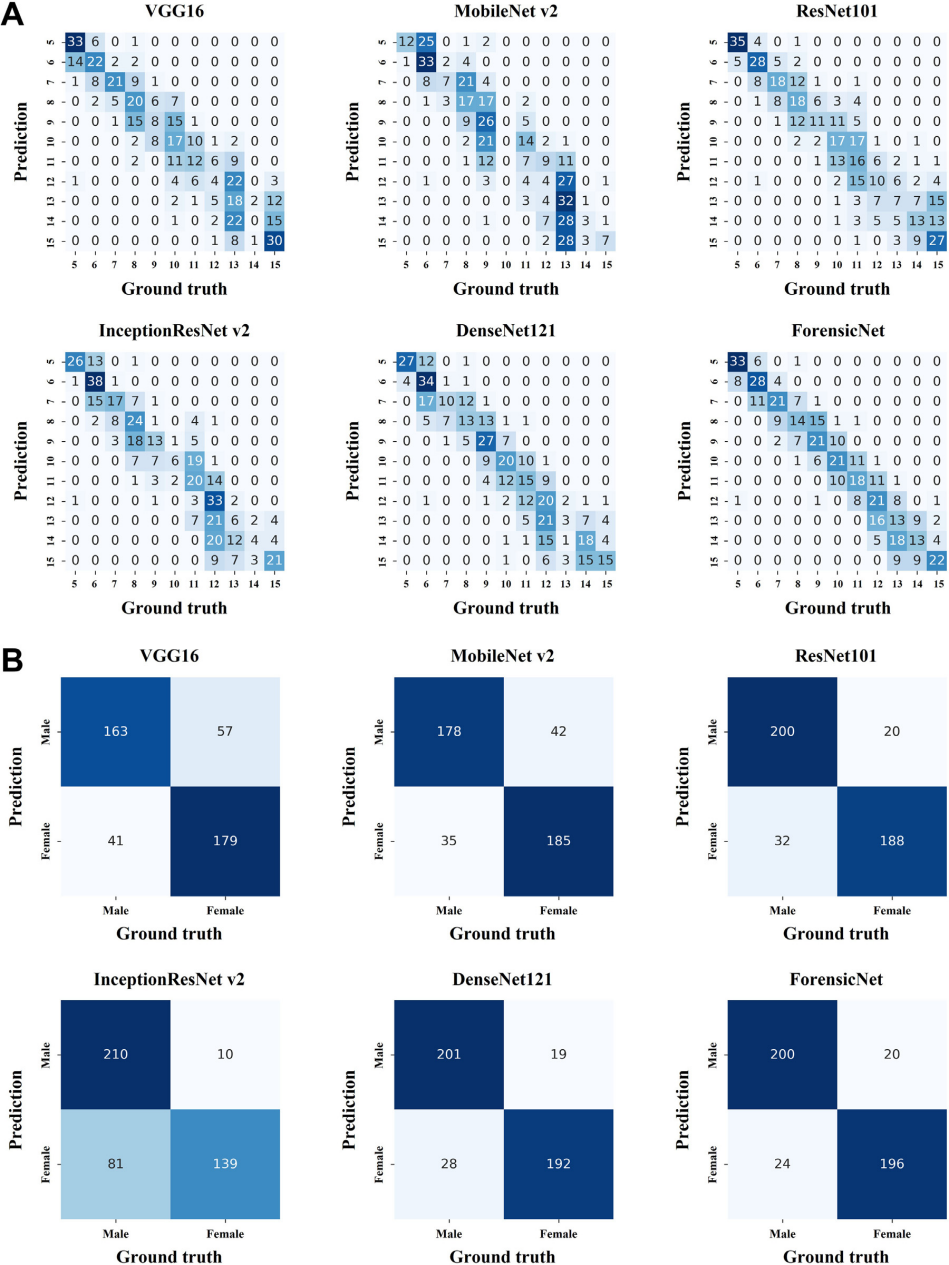
(A) Confusion matrices for chronological age estimation across different networks. Darker blue indicates higher frequency; color intensity reflects classification accuracy. (B) Confusion matrices for sex classification across different networks. Darker blue indicates higher frequency; color intensity reflects classification accuracy.

As illustrated in Figure 2A, the confusion matrices of the evaluated networks revealed varying classification patterns for chronological age estimation. ForensicNet demonstrated a more balanced distribution of false positives in relation to true positive cases, indicating consistent performance across age categories. While InceptionResNet v2 achieved the highest number of true positives (n = 38) for a single age group (6 years old), ForensicNet attained the highest overall sum of true positive classifications (n = 225) across all age groups. Building on this, Figure 2B shows that ForensicNet also achieved the highest number of true positive classifications for females (n = 196) among the evaluated networks, along with the fewest false positives (n = 24). For male classification, InceptionResNet v2 exhibited the best performance, with 210 true positives and only 10 false positives.

Regarding the area under the curve, values ranged from 0.864 to 0.973, with ForensicNet attaining the highest value (Supplemental Figure S2A). In Supplemental Figure S2B, Brier score distributions for sex classification across different networks ranged from 0.07 to 0.15, with ForensicNet achieving the lowest Brier score, indicating superior overall calibration and predictive accuracy.

When comparing ForensicNet's performance metrics for both chronological age estimation and sex classification with and without CBAM, the inclusion of CBAM yielded superior results. Specifically, the CBAM-enhanced model achieved lower MAE and MD, higher R^2^ in age estimation and improved ACC, SPE and SEN in sex classification (supplemental Table S2).

Upon evaluation of the optimal weighting configuration for the multitask loss function in ForensicNet, the best overall performance for chronological age estimation was achieved with *α* = 0.3 and *β* = 0.7, while the highest performance for sex classification was observed with *α* = 0.1 and *β* = 0.9 (Supplemental Table S3).

**Fig. 3:**
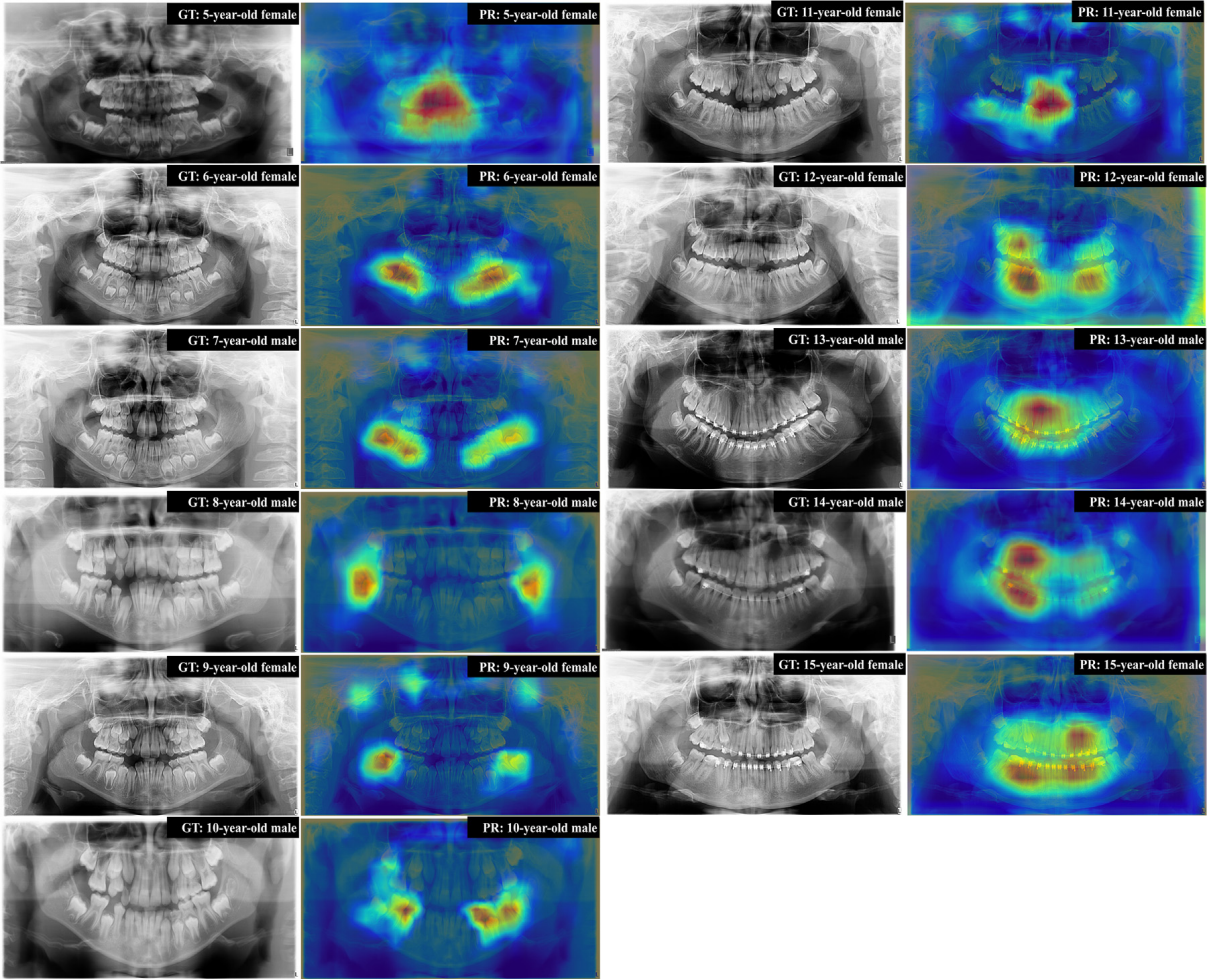
Representative successful estimations and corresponding Grad-CAM visualisations from ForensicNet. GT, ground truth; PR, prediction.

When evaluating the heatmaps generated by Grad-CAM, successful cases of chronological age estimation and sex classification (Figure 3) demonstrated concentrated attention in the dentate region across deciduous, mixed and permanent dentitions. In contrast, unsuccessful cases (Supplemental Figure S3) exhibited more diffuse attention across various dentomaxillofacial structures, including the nasal cavity, mandible and cervical spine.

## Discussion

This study proposed a multitask DL model based on ForensicNet^17^ to simultaneously estimate chronological age and classify sex from panoramic radiographs of young Brazilian individuals. The model outperformed five benchmark architectures without age-specific adaptation or multitask learning by leveraging shared feature representations. The integration of CBAM enabled ForensicNet to focus on anatomically relevant features, such as permanent/deciduous teeth and alveolar bone morphology, supporting the anatomical plausibility of its decisions. Grad-CAM heatmaps confirmed its consistent attention to meaningful regions, enhancing interpretability and clinical relevance. In paediatric samples aged 5-10 years, ForensicNet mainly focused on deciduous dentition; from 11 to 15 years, heatmaps shifted to permanent dentition and showed activity in areas of dental brackets or wires, which may indirectly indicate chronological age.^26^ ForensicNet thus captures both anatomical and nonanatomical features relevant to age and sex estimation, underscoring its robustness and interpretability.

The use of AI in forensic dentistry is still emerging, but its potential applications are considerable.^6^^,^^27^ Deep learning models can improve accuracy and efficiency by assisting in the analysis of large volumes of dental images, reducing observer-dependent variability and automating tasks that traditionally require substantial manual effort.^6^ In addition, AI can enhance radiographic quality, support age estimation, assist in matching antemortem and postmortem dental records, contribute to bite-mark comparisons and even aid in the generation of 3D reconstructions for identification.^6^ These advantages reinforce the relevance of integrating AI as a complementary tool capable of optimizing workflows and improving the consistency of forensic assessments.

However, despite these promising advances, important limitations and cautions must be considered. AI models may reflect or amplify biases inherent to their training datasets, which can affect their performance across different populations and potentially lead to misclassification.^6^ Issues related to transparency and interpretability also remain a challenge, as many deep learning architectures do not disclose the workflow, making it difficult to fully understand the basis of their predictions, which is particularly relevant in forensic contexts.^6^ Privacy, data security and the ethical origin of training data are additional concerns that require careful attention. For these reasons, AI systems should be implemented with appropriate oversight and continuous validation, always functioning as support tools rather than replacements for forensic experts, whose clinical and contextual judgment remains essential for the reliable and responsible use of these technologies.

Despite the overall superior performance of ForensicNet, several instances of misestimation were observed, as illustrated in Supplemental Figure S3. Grad-CAM heatmaps indicated that, in many of these error cases, ForensicNet attended to less informative or ambiguous regions, such as indistinct bone structures or diffusely distributed areas of sparse activation, rather than consistently focusing on the deciduous or permanent dentition. For example, in patients aged 9-12 years, where mixed dentition is present, the overlap of developmental stages between deciduous and permanent teeth may contribute to increased uncertainty in age estimation. Furthermore, misestimated cases frequently exhibited irregular dental characteristics, such as delayed eruption patterns or dental crowding, which deviate from the age-typical anatomical patterns represented in the training data. Also, ForensicNet presented the higher MD, which indicates that, although the overall performance metrics were strong, isolated cases showed larger discrepancies between predicted and actual ages. This suggests that the model may be more sensitive to certain outlier presentations or atypical anatomical patterns.

ForensicNet was evaluated in comparison with prior DL approaches for chronological age estimation and sex classification using panoramic radiographs of paediatric and adolescent populations. Guo et al.^12^ and Bu et al.^13^ utilised large-scale datasets comprising over 10,000 images; however, their models were restricted to single-task prediction and demonstrated diminished performance in younger cohorts. For example, Bu et al.^23^ reported a lower accuracy for sex classification in minors (82.64%) relative to adults (90.97%), highlighting the limitations in model generalizability to pediatric populations. Other investigations achieved competitive age estimation performance, MAEs of 0.56 and 0.72, respectively, using comparatively smaller datasets, but did not implement multitask learning strategies.^14^^,^^15^ Another previous study focused exclusively on sex classification in a large pediatric cohort, without integrating chronological age estimation.^16^ In contrast, ForensicNet employs a multitask learning architecture that concurrently estimates chronological age and classifies sex through task-specific attention mechanisms implemented via CBAM. The model was trained and validated on a dataset characterised by a balanced distribution of chronological age and sex, ranging from 5 to 15 years, thereby reducing potential biases associated with data imbalance. Consequently, ForensicNet achieved robust and comparable performance for both chronological age estimation and sex classification in panoramic radiographs of Brazilian children and early adolescents.

The superior performance of ForensicNet across multiple metrics can be related to the design and intended application of the architecture. ForensicNet was developed specifically for forensic and dental imaging, enabling it to extract domain-relevant anatomical patterns that enhance chronological age estimation and sex classification.^17^ In contrast, MobileNet v2^22^ and InceptionResNet v2^24^ are general-purpose architectures initially created for broad image recognition tasks, which influences how they perform in forensic applications. MobileNet v2 is a lightweight model optimised for computational efficiency through simplified convolutional layers, which may explain its lower mean deviation but also its limitations in capturing more complex structural features.^22^ InceptionResNet v2, conversely, is a deep, high-capacity network that combines Inception blocks with residual connections, allowing it to learn highly detailed representations, reflected here in its higher specificity for sex classification.^24^ It is important to recognize that different convolutional neural network (CNN) architectures prioritize different aspects of learning, such as efficiency, depth or domain specificity and these intrinsic differences naturally lead to variations in performance across forensic tasks.

In the present study, the inclusion of Brazilian individuals reflects the aim to evaluate the performance of ForensicNet in an ethnically distinct group compared with the original South Korean cohort in which the model was first developed and validated.^17^ Assessing the model in such a distinct population provides an opportunity to test its generalizability and robustness when applied beyond the demographic characteristics of the initial dataset. This approach strengthens the external validity of the findings and contributes to understanding how AI-driven forensic tools may behave in multiethnic contexts. Additionally, a recent study applying a CNN-based model for dental age estimation in an Indonesian population within the same age range (5-15 years) demonstrated accurate performance but also highlighted differences in accuracy across age groups, particularly in younger individuals.^28^

The present study has some limitations that are important to acknowledge for offering directions for future research. First, the dataset used in this study comprises panoramic radiographs from individuals aged 5-15 years, which may restrict the model’s applicability to other age groups. Future studies should consider expanding the age range to improve the model’s utility across a broader demographic spectrum. Second, the data were collected from a single institution and represent individuals from the Brazilian population. While this provides a consistent foundation for model development, it may limit generalizability to other ethnicities, geographic regions or forensic scenarios involving deceased individuals. Given that dental and craniofacial development can vary among populations, future work should incorporate multi-institutional and ethnically diverse datasets to assess cross-population robustness. Third, although Grad-CAM was utilised to enhance model interpretability, some visualisations revealed attention in less informative regions, such as the corners of images or areas of diffuse activation. This indicates that the model retains certain 'black box' characteristics, underscoring the need for further efforts in explainable AI to improve clinical transparency and trust.^29^ Addressing these limitations in future research will be essential for developing more robust, equitable and clinically interpretable AI tools for forensic and diagnostic applications.

In conclusion, ForensicNet demonstrated strong effectiveness as a multitask deep learning model for the simultaneous estimation of sex and chronological age from panoramic radiographs of Brazilian individuals aged 5-15 years. The model’s performance was enhanced through the use of a balanced dataset, the incorporation of convolutional block attention modules and a weighted multitask loss function, all of which contributed to the optimisation of both predictive tasks. Interpretability analyses revealed that the model consistently attended to anatomically and clinically relevant regions, underscoring its potential utility in forensic and diagnostic contexts. These findings support the reliability, robustness and clinical interpretability of ForensicNet for age and sex estimation in pediatric populations. Future research should aim to improve the model’s generalizability by incorporating larger, multicentre and ethnically diverse datasets, as well as exploring hybrid architectures that integrate convolutional neural networks, transformers and diffusion-based models.

## Funding

This study has received funding by the National Research Foundation of Korea (NRF) grant funded by the Korean government (MSIT) (No.2023R1A2C200532611), Technology Innovation Program (or Industrial Strategic Technology Development Program-Advanced Biomaterials) (RS-2025-14322975) funded By the Ministry of Trade Industry & Energy (MOTIE), University of Campinas (UNICAMP), through its International Office (DERI), under call 64/2024 – DERI – Santander Program for International Mobility: Strategic Partnerships and Coordenação de Aperfeiçoamento de Pessoal de Nível Superior - Brasil (CAPES) - Finance Code 001.

## Author contributions

*Concept and design:* All authors.

*Acquisition, analysis, or interpretation of data:* All authors.

*Drafting of the manuscript:* All authors.

*Supervision:* Won-Jin Yi and Min-Suk Heo.

## Conflicts of interests

None disclosed.

## Appendix Supplementary materials

Image, application 1
