# Supplementary material for: Multi-Task Deep Learning for Sex and Age Estimation from Panoramic Radiographs in a Brazilian Young Population
Source: Int Dent J. 2026 Jan 27;76(2):109381. doi: 10.1016/j.identj.2025.109381 (PMC12865573; doi:10.1016/j.identj.2025.109381)
Supplement: Supplementary file 1 [file mmc1.docx]

**Supplementary materials**

***Training Setup***

The multi-task DL framework was trained for 200 epochs using a mini-batch size of 16. To enhance model generalization, data augmentation was applied during training, including random rotations in the range of -10° to 10°, as well as horizontal and vertical shifts of up to ±10% of the image dimensions. Network optimization was performed using the Adam optimizer with parameters β_0_=0.9 and β_1_=0.999. The initial learning rate was set to 10^-3^ and was reduced by half whenever the validation loss plateaued for 25 consecutive epochs, with a minimum threshold of 10^-6^. The implementation was carried out in Python3 using the Keras library with a TensorFlow backend. All experiments were conducted on an NVIDIA TITAN RTX GPU with 24 GB of memory.

***Performance Evaluation***

To further analyze classification performance, confusion matrices were independently interpreted to evaluate the classification performance of each model for both sex estimation and chronological age estimation. For sex classification, predicted labels were compared against the true sex labels from the test dataset. For age estimation, age predictions were categorized into discrete age classes ranging from 5 to 15 years and compared to the corresponding true ages. Each confusion matrix tabulates true positives, true negatives, false positives, and false negatives, allowing a detailed assessment of accuracy and misclassification patterns. All confusion matrices were derived from the final model predictions on the held-out test set to ensure an unbiased evaluation of generalization performance.

In addition, to provide a detailed, age-specific performance analysis, mean absolute error was calculated separately for each individual age (from 5 to 15 years) across all classification networks. This fine-grained evaluation offers quantitative insight into the reliability of model predictions at specific developmental stages, which is particularly relevant in forensic contexts where age estimation accuracy can be critical.

As previously described, ForensicNet was initially designed with the CBAM to enhance its capacity to extract discriminative features from dental panoramic radiographs. To evaluate the specific contribution of this attention mechanism, ablation experiments were conducted by implementing a variant of ForensicNet without CBAM, and performance outcomes for both age and sex estimation were compared between the two versions.

To investigate the impact of task prioritization within the multi-task DL framework, ablation experiments were conducted in which ForensicNet was trained multiple times using varying combinations of the weighting parameters *α* and *β*. These experiments aimed to assess how different emphasis levels on the age and sex estimation tasks affected overall performance.


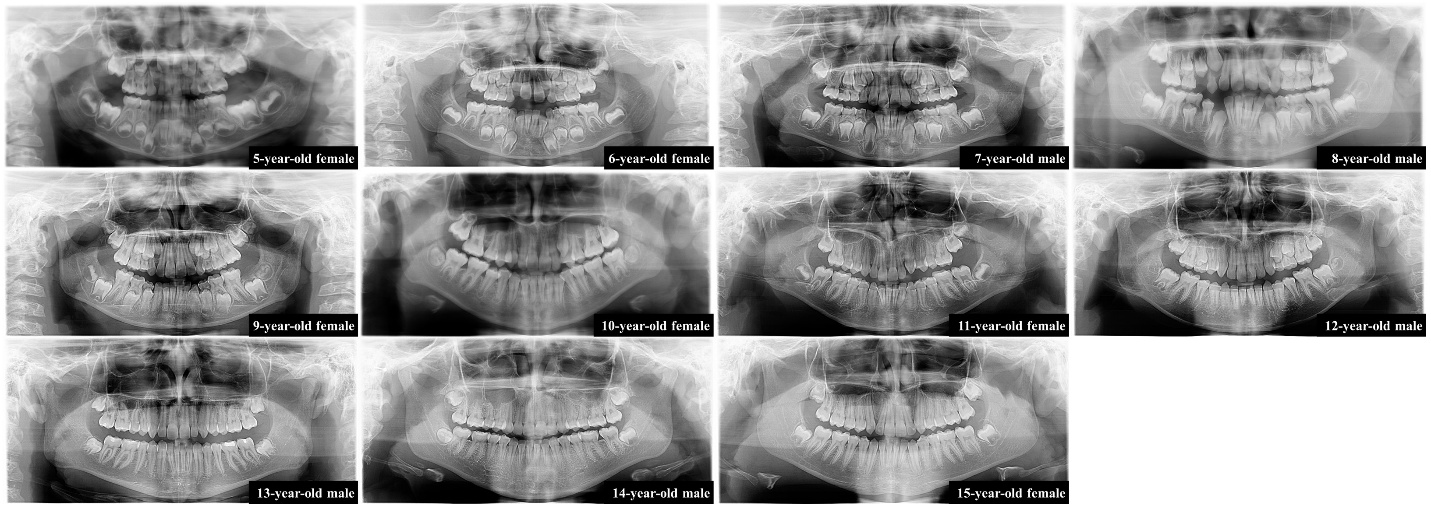


**Figure S1.** Samples of panoramic radiographs from male and female individuals aged 5–15 years from the Brazilian population.


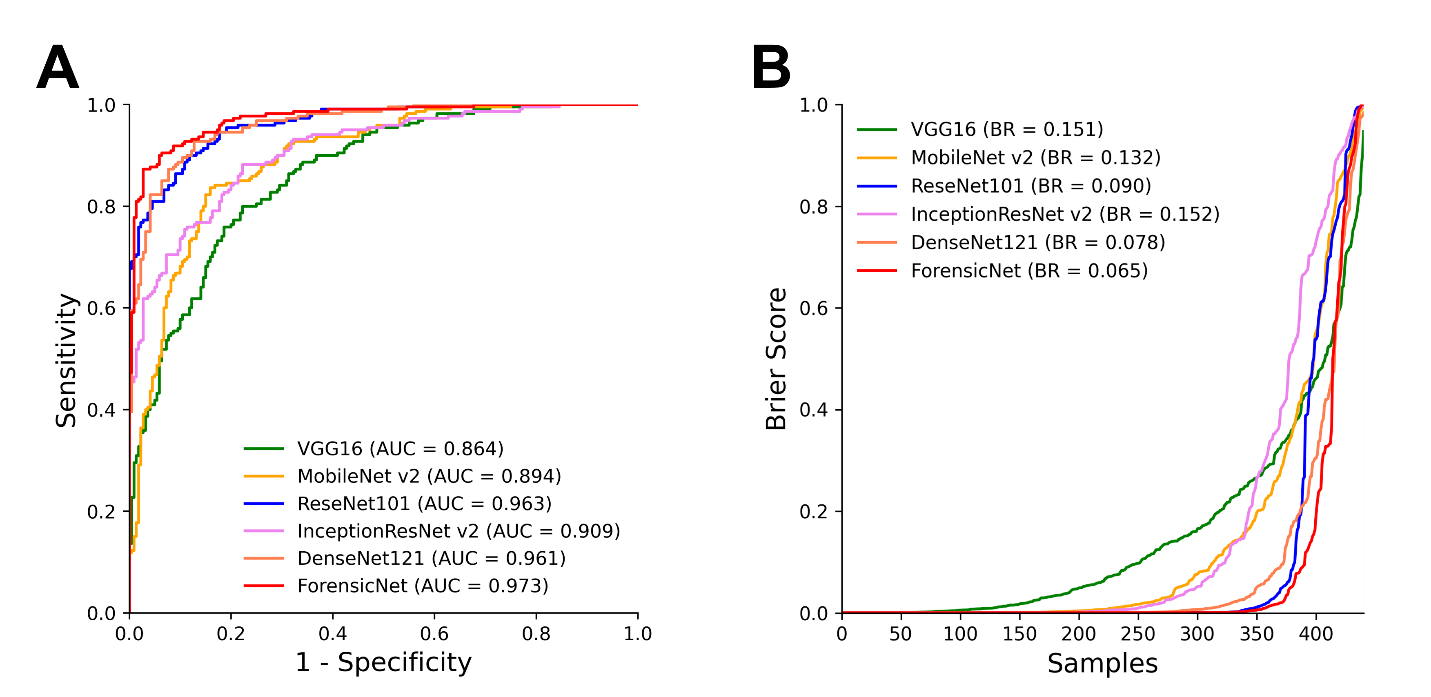


**Figure S2.** Sex classification performance across networks: (A) ROC curves with area under the curve values; (B) Brier score distribution.


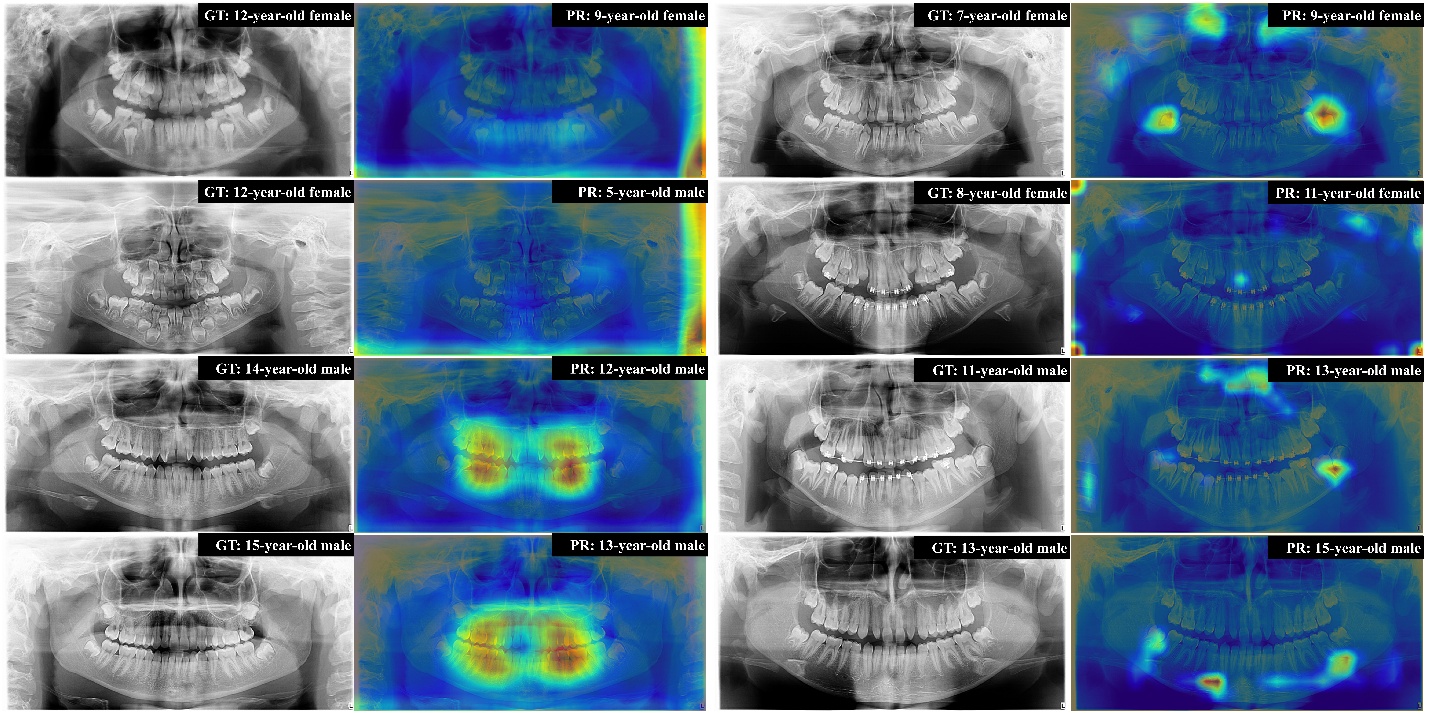


**Figure S3.** Representative misestimations and corresponding Grad-CAM visualizations from ForensicNet. GT: ground truth; PR: prediction.

**Table S1.** Mean absolute errors of deep learning networks for chronological age estimation, categorized into age groups ranging from 5 to 15 years

| Deep learning network | 5 | 6 | 7 | 8 | 9 | 10 | 11 | 12 | 13 | 14 | 15 |
| --- | --- | --- | --- | --- | --- | --- | --- | --- | --- | --- | --- |
| VGG16 | 0.23 | 0.50 | 0.53 | 0.73 | 0.85 | 0.75 | 1.03 | 1.30 | 0.98 | 1.13 | 0.50 |
| MobileNet v2 | 0.90 | 0.28 | 0.93 | **0.70** | 0.48 | 1.15 | 1.45 | 1.23 | **0.28** | 1.20 | 1.63 |
| ResNet101 | **0.18** | 0.35 | 0.65 | 0.85 | 0.88 | 0.73 | 0.80 | 1.18 | 1.33 | 1.03 | **0.48** |
| InceptionResNet v2 | 0.40 | 0.50 | 0.60 | 0.73 | 0.88 | 1.05 | 0.63 | **0.38** | 1.13 | 1.40 | 1.10 |
| DenseNet121 | 0.38 | **0.18** | 0.78 | 0.88 | **0.35** | **0.53** | 0.73 | 0.80 | 1.15 | 1.05 | 1.10 |
| ForensicNet | 0.23 | 0.30 | **0.50** | 0.73 | 0.53 | **0.53** | **0.58** | 0.73 | 0.73 | **0.80** | 0.68 |

Bold values indicate the best performance for each metric across networks.

**Table S2.** Performance of the ForensicNet network for both chronological age estimation and sex classification without and with a convolutional block attention module (CBAM)

| CBAM | Chronological age | | |  | Sex | | |
| --- | --- | --- | --- | --- | --- | --- | --- |
|  | MAE (↓) | MD (↓) | R^2^ (↑) |  | ACC (↑) | SPE (↑) | SEN (↑) |
| Without | 0.68±0.82 | **5.32** | 0.89 |  | 0.88 | 0.89 | 0.87 |
| With | **0.57±0.71** | 6.43 | **0.92** |  | **0.90** | **0.91** | **0.89** |

MAE, mean absolute error (±standard deviation); MD, maximum deviation; R², coefficient of determination; ACC, accuracy; SPE, specificity; SEN, sensitivity.

Upward (↑) and downward (↓) arrows indicate the desired direction of improvement.

Bold values indicate the best performance for each metric across networks.

**Table S3.** Performance of the ForensicNet network for both chronological age estimation and sex classification according to weights for 𝛼 and 𝛽 in the total weighted multi-task loss

| Weight | |  | Chronological age | | |  | Sex | | |
| --- | --- | --- | --- | --- | --- | --- | --- | --- | --- |
| $\alpha$ | $\beta$ |  | MAE (↓) | MD (↓) | R^2^ (↑) |  | ACC (↑) | SPE (↑) | SEN (↑) |
| 0.1 | 0.9 |  | 0.70±0.82 | 5.30 | 0.88 |  | 0.**92** | 0.88 | 0.**96** |
| 0.2 | 0.8 |  | 0.73±0.80 | 6.28 | 0.88 |  | 0.89 | 0.85 | 0.93 |
| 0.3 | 0.7 |  | **0.57±0.71** | 6.43 | **0.92** |  | 0.90 | 0.91 | 0.89 |
| 0.4 | 0.6 |  | 0.65±0.78 | 5.35 | 0.90 |  | 0.86 | 0.80 | 0.92 |
| 0.5 | 0.5 |  | 0.74±0.85 | 6.26 | 0.87 |  | 0.88 | 0.86 | 0.89 |
| 0.6 | 0.4 |  | 0.71±0.84 | 6.29 | 0.88 |  | 0.83 | 0.74 | 0.92 |
| 0.7 | 0.3 |  | 0.73±0.89 | **5.25** | 0.87 |  | 0.85 | 0.86 | 0.84 |
| 0.8 | 0.2 |  | 0.72±0.83 | 6.28 | 0.88 |  | 0.85 | 0.84 | 0.86 |
| 0.9 | 0.1 |  | 0.85±1.01 | 6.15 | 0.82 |  | 0.55 | **1.00** | 0.11 |

MAE, mean absolute error (±standard deviation); MD, maximum deviation; R², coefficient of determination; ACC, accuracy; SPE, specificity; SEN, sensitivity.

Upward (↑) and downward (↓) arrows indicate the desired direction of improvement.

Bold values indicate the best performance for each metric across networks.
